# Diseases of Children

**Published:** 1879-07

**Authors:** 


[From "Hall's Journal of Health" for Sept., 1876.]

### BOWEL COMPLAINTS OF CHILDREN.

It must not be supposed that we mean only the state of flux or diarrhœa when we speak of children's bowel complaints. That condition can not be called disease any more than other symptoms which often precede it. Bowel complaint is as likely to be starvation as anything else, and as often manifests itself by a *lack* of fluid in the discharges from the bowels as in any other way. We believe we state the simple truth when we say that of the five or six hundred little ones who have died each week in New York city, beginning with June last, nineteen in twenty have died from innutrition—that is, starvation.

We do not mean by this to assert that the little ones have not been supplied with food. This is not the general fault. Most children, even those of poor parents, have sufficient food to sustain life offered to them each day. It is not always perfect food ; it is, indeed, very often, quite imperfect ; but it would ordinarily be adequate to the support of life if the power to convert it into blood and tissue existed in the child. Unfortunately this power is often lacking, and in such cases the strength imparted bears no relation to the amount and quality of food taken into the stomach. In fact, where the power to convert is lacking, food of any sort may easily become an element of weakness rather than an element of strength.

It is not always possible to say just *why* an infant suffers from innutrition. We have seen cases in which, after all other explanations failed, it was clearly shown to be an hereditary weakness of the gastric fluids, dating back, perhaps, through three or four generations of constipated dyspeptics. The malady manifests itself in various ways. Perhaps the child suffers pain after eating ; or the bowels swell ; or the discharges are green and watery and offensive, or are hard and whitish, like cheese ; or contain particles of mucus, resembling melted glue or mucilage ; or the child seems stupid and sleepy, and is roused with difficulty ; or the flesh grows soft and flabby, and loses its plumpness and wholesome color. Later, the brain becomes affected, and the pulse intermits.

All these are dangerous symptoms, and admit of only one interpretation. It is this : the food does not make blood, does not supply material for growth. It is as much a stomach-complaint as a bowel-complaint. It is faulty digestion, or no digestion at all. The food accomplishes no useful purpose, and the child starves to death.

In almost all such cases the last days or hours are attended with a loose discharge from the bowels. This is not the disease ; it is only nature's method of trying to relieve the disease. The alimentary canal has become clogged with undigested food, and nature pours out her mucus fluids in her efforts to rid the intestines of their burden. This fluid is her lubricator, that is all. But observe how the average doctor treats this well-meant act. He says this discharge is dangerous, and must be stopped. So he gives opium in some form, combined with astringents, perhaps, and the points of exit for this salutary fluid are sealed up. This sudden suppression of any secretion is always dangerous. It will stop of itself if left to itself, and if other conditions are fulfilled. But when you suddenly seal up the pores of this long intestinal membrane, you inevitably send these active fluids to the brain, there to induce pressure, congestion, and death.

Now, assuming that bowel complaint of children—whether it shows [illegible] by constipation, or by fluid evacuations, or by pain, or by curd-like scharges, or in any other way—is innutrition, which means starvation, what is the remedy? We reply, beyond a doubt the use of such means as will strengthen the assimilative power; the employment of methods calculated to add potency to the gastric fluids. If the stomach lacks the power to emulsify its contents, it must be aided in its labors. If the liver fails to perform its part of the work, it must be encouraged and sustained. If the absorbants neglect to appropriate the nourishing fluids discharged upon them, they must be stimulated up to honest work.

If the child gets its food-supply from the mother's breast, her food and her habits and her health must be looked after. Perhaps her diet is bad; or there may be a drain or disease about her which she treats lightly, or of which she is unconscious. With the mother set right, nursing infants usually fall into healthful conditions. If brought up by hand, the case is different. Then there must be knowledge concerning the chemistry of foods, or success in rearing children will be largely a thing of luck. With the food question accurately and intelligently settled, if there is still indigestion in any form, whether it manifests itself by vomiting or purging, or constipation, or colic, or stupor, or sleeplessness, or paleness, or flabbiness—the one essential need is *the added power of assimilation*, and this power must be conferred by such gentle and safe means as medical skill would readily suggest.

---

If we were asked, What harmless form of medicine would you prescribe for the ordinary forms of innutrition, starvation, stomach and bowel-complaint of children? we should be compelled to answer that the best assimilative article we have ever used is what is known as Van Deren's prescription. The effect of this remedy is unquestionably to increase the solvent power of the gastric fluids. Its great virtue lies in the fact that through its aid the food taken by the little one is much more readily and rapidly converted into blood. With perfect nutrition, the infant's hold upon life is exceedingly strong. With faulty digestion it is next to impossible to carry a child through the first five years of life. It would be a wise act for all mothers to keep a package of these little pellets at hand, to use freely in all cases of indigestion, so that the little ones may have an appetite for food, and may possess the power to employ food for the purpose of sustaining and building up the slight frame-work of life.

---

"ALL THE YEAR ROUND."

"Van Deren's Remedy" is not for summer diseases of children alone, but will be found the safest and best remedy for the various stomach and bowel troubles to which young children are liable at all seasons of the year. The editor of HALL'S JOURNAL OF HEALTH has prescribed it in his own practice for years, and his opinion as expressed above, is the result of long experience and careful observation. One great advantage of "Van Deren's Remedy" is that it can be easily administered. It [illegible] made in the form of small homœpathic sugar granules.

DIRECTIONS.—To a child under six months *four* of these little pelle[illegible] should be given every two, three, or four hours, depending on the urgency of the symptoms. To a child between six and twelve months *eight* pellets should be given as above. To a child between one and two years old, *twelve* pellets may be given at a dose. Over two years *sixteen* pellets.

**Price 50 cents a box.** Sent free by mail on receipt of price.

HALL & RUCKEL, Wholesale Druggists,

